# Correlates of HIV self-testing among female sex workers in China: implications for expanding HIV screening

**DOI:** 10.1186/s40249-020-00765-5

**Published:** 2020-10-22

**Authors:** Cheng Wang, Ya-Jie Wang, Joseph D. Tucker, Ming-Zhou Xiong, Hong-Yun Fu, M. Kumi Smith, Wei-Ming Tang, Jason J. Ong, He-Ping Zheng, Bin Yang

**Affiliations:** 1 Dermatology Hospital of Southern Medical University, Guangzhou, Guangdong China; 2 Southern Medical University Institute for Global Health and Sexually Transmitted Diseases, Guangzhou, Guangdong China; 3 University of North Carolina Project-China, Guangzhou, Guangdong China; 4 Faculty of Infectious and Tropical Diseases, London School of Hygiene and Tropical Medicine, London, UK; 5 Institute for Global Health and Infectious Diseases, School of Medicine, University of North Carolina At Chapel Hill, Chapel Hill, USA; 6 Division of Community Health and Research, Eastern Virginia Medical School, Norfolk, VA USA; 7 Division of Epidemiology and Community Health, University of Minnesota Twin Cities, Minneapolis, USA; 8 Central Clinical School, Monash University, Victoria, Melbourne Australia

**Keywords:** HIV, Self-testing, Female sex workers, China

## Abstract

**Background:**

Human immunodeficiency virus (HIV) self-testing may help improve test uptake among female sex workers. China has implemented many HIV self-testing programs among men who have sex with men, creating an opportunity for promotion among female sex workers. However, there is a limited literature on examining HIV self-testing among female sex workers. This study aimed to examine HIV self-testing experiences and its determinants among female sex workers in China.

**Methods:**

A venue-based, cross-sectional study was conducted among Chinese female sex workers in 2019. Participants completed a survey including social-demographic characteristics, sexual behaviors, and HIV self-testing history, the distribution of which were analyzed using descriptive analysis. Multivariable logistic regression was conducted to identify associations with HIV self-testing.

**Results:**

Among 1287 Chinese female sex workers, 1072 (83.3%, 95% confidence interval [*CI*] 81.2–85.3%) had ever tested for HIV, and 103 (8.0%, 95% *CI* 6.6–9.6%) had ever used HIV self-testing. More than half reported that the self-test was their first HIV test (59.2%, 61/103), around one-fifth reported HIV self-testing results influenced the price of sex (21.4%, 22/103). A minority of individuals reported ever experiencing pressure to undertake HIV self-testing (6.8%, 7/103). After adjusting for covariates, HIV self-testing was positively associated with receiving anal sex in the past month (adjusted odds ratio [*aOR*] = 2.2, 95% *CI* 1.4–3.5), using drugs before or during sex (*aOR* = 2.8, 95% *CI* 1.8–4.5), injecting drugs in the past 6 months (*aOR* = 2.6, 95% *CI* 1.2–6.0), being diagnosed with other sexually transmitted infections (*aOR* = 1.6, 95% *CI* 1.0–2.5), tested for other sexually transmitted infections in the past six months (a*OR* = 3.4, 95% *CI* 2.1–5.5), ever tested in the hospital (*aOR* = 3.4, 95% *CI* 2.0–5.6), and ever tested in the community (*aOR* = 1.5, 95% *CI* 1.2–1.9).

**Conclusions:**

Our findings suggest that HIV self-testing could expand overall HIV testing uptake, increase HIV testing frequency, reach sub-groups of high-risk female sex workers and has limited potential harms among female sex workers. HIV self-testing should be incorporated among Chinese female sex workers as a complement to facility-based HIV testing services.

## Background

An estimated 85% of all HIV infections are transmitted through heterosexual sex worldwide [1]. Female sex workers are at high risk of human immunodeficiency virus (HIV) and other sexually transmitted infections (STI) acquisition [2]. The World Health Organization recommends frequent HIV testing in female sex workers to increase engagement in HIV prevention services and reduce risk of onward transmission [3]. However, HIV testing uptake remains low among female sex workers in low- and middle- income countries (LMIC) [4, 5]. In China, studies suggest that approximately half of female sex workers remain unaware of their HIV serostatus [6, 7].

Although facility-based HIV testing have helped increase testing coverage among female sex workers in China [7, 8], many barriers persist. These include concerns about confidentiality of status [9], lack of privacy [7], fear of social stigma and condemnation [10], lack of health care providers and inconvenient testing systems [11].

Self-testing for HIV may help improve test uptake among female sex workers. Self-testing is the process whereby a person collects a specimen, performs the test, and interprets the result themselves. Studies in female sex workers in Uganda [12], Kenya [13], Malawi [14], and Zambia [15] have shown that HIV self-testing can increase the frequency of HIV testing and safer sex practices, decrease stigma associated with HIV testing, provide a user-friendly, rapid, accurate and private setting of testing, as well as an opportunity for decentralized HIV testing and alternative service delivery models. However, there have been few studies examining HIV self-testing among female sex workers in countries outside of sub-Saharan Africa, including China [16–18]. Currently, a total of 59 countries globally have policies supporting using HIV self-testing among key populations [19]. In China, highly sensitive and specific HIV self-test kits are available through community-based organizations or e-commerce platforms [11]. China has implemented many HIV self-testing programs and gained experience among men who have sex with men (MSM) [20, 21], creating an opportunity to promote HIV self-testing among female sex workers. The aim of this study was to examine HIV self-testing experiences and its determinants among female sex workers in China.

## Methods

### Study design and participants

A venue-based, cross-sectional study was conducted in eight cities (Beijing, Tianjin, Shenzhen, Kunming, Jiaozhou, Yunfu, Xiangyang and Longnan) within seven provinces in China between August 17 and October 17, 2019. These eight cities were selected based on local capacity and the availability of ongoing public health outreach programs for female sex workers.

We partnered with eight local female sex workers community-based organizations (CBO) in those eight cities with experience of conducting female sex workers outreach programs including condom promotion, sexual health education, HIV and syphilis rapid testing and counseling, and linkage to care (accompaniment to clinical services for infected individuals).

Prior to this study, a mapping of the sex work venues was performed by local CBO in each study site according to geographic area and type of venue. A convenience sampling method was used to recruit female sex workers in selected venues in each city. We categorized the sex work venues into high tier and low tier based on the clientele’s socioeconomic status [22]. Low tier venues include foot bathing shops, hair salons or barber shops, massage parlors, roadside restaurants, roadside shops, guesthouses, streets or public outdoor places. High tier venues include karaoke bars, hotels, sauna, and nightclub. At each site, at least 30% of participants were low-tier sex workers.

The inclusion criteria for this study were as follows: born biologically as a female; aged 18 or above; exchanged sex at least once for money/goods in the past three months; willing to participate and complete the survey.

### Data collection

The survey questionnaire was created on Wenjuanxing (Changsha Haoxing Information Technology Co., Ltd., Changsha, China) based on discussions with local CBO stakeholders, policy makers and international HIV experts. We also piloted the survey with 50 volunteer female sex workers. The purpose of this formative research was to ensure the survey was simple to complete and consistent with our written survey content. This pilot data was not included in the final analysis.

In the formal survey, each questionnaire was self-administered by eligible participants with the help of outreach workers. Participants would receive United States dollars (USD) 2 on completion of the study. The questionnaires submitted within five minutes were deemed invalid based on our pilot testing prior to the study that a minimum time of five minutes were required to respond all the survey items.

### Measures

#### Social-demographic and sexual behavior characteristics [23]

Social-demographic and sexual behavior characteristics included: age, marital status, place of residence, annual income, education, time of providing commercial sex in the current location, number of cities worked for selling sex, number of clients served in the past month, charge for vaginal sex, whether condoms were used consistently when engaged in commercial sex in the past month, illicit substance use, STI testing history. Consistent condom use in the past month was defined as always using a condom during commercial sex.

#### Self-testing history for HIV

Self-testing history for HIV included location where the kit was obtained, self-test results, post-test healthcare seeking behaviors, change in testing frequency after the first use of a self-test kit, experienced pressure from self-testing, whether giving or selling a self-test kit to a client, influences of performing self-testing on sex exchange. Categories of pressure included physical violence, threats of violence, verbal abuse, psychological pressure, excessive control of activities, withholding of household resources, and threats to end a relationship [23].

### Statistical analysis

Descriptive analysis was conducted to describe the distribution of the sample regarding background characteristics, substance use, sexual behaviors, HIV and syphilis testing.

Univariable and multivariable logistic regression was conducted to explore socio-demographic and behavioral variables associated with HIV self-testing. In the multivariable model we adjusted for age, legal marital status, educational attainment, and annual income. All analyses were conducted on SAS (V9.2, SAS Institute Inc., Cary, NC).

### Ethic review

This study was approved by the Dermatology Hospital of Southern Medical University (2019017). A verbal informed consent was obtained from all the participants who agreed to participate in this study.

## Results

Overall, 1443 women met the inclusion criteria. Eighty-one individuals declined to participant the study, and 77 completed the questionnaire less than five minutes. Finally, a total number of 1287 women completed the survey. Among those participants, 1072 (83.3%) had ever tested for HIV, and 103 (8.0%) had ever used HIV self-testing.

### Study participants characteristics

Most participants were between 18 and 35 years old (53.8%), from low tier venues (57.3%), married (43.8%), had a junior high school degree (45.2%), and had an annual income between USD 5001 and USD 14 000 (56.3%). The majority were employed (54.4%), residing in the province where the study was done (51.4%) and working in current location over one year (50.51%). The socio-demographic characteristics of respondents who ever used HIV self-testing were comparable to women who never used HIV self-testing (Table 1).

**Table 1:** Social demographic characteristics among female sex workers, a cross-sectional survey in China, 2019

Characteristics	Total	HIV testing, *n* (%)		
		Self-tester	Facility-tester	Non-tester
Total*	1287	103 (8.0)	969 (75.3)	215 (16.7)
Age				
18–25	271 (22.5)	13 (13.4)	221 (24.2)	37 (19.2)
26–35	376 (31.3)	28 (28.9)	269 (29.5)	79 (40.9)
36–45	312 (25.9)	32 (33.0)	221 (24.2)	59 (30.6)
> 45	244 (20.3)	24 (24.7)	202 (22.1)	18 (9.3)
Workplace				
High tier	549 (42.7)	49(47.6)	428 (44.2)	72 (33.5)
Low tier	738 (57.3)	54 (52.4)	541 (55.8)	143 (66.5)
Legal marital status				
Never married and not cohabiting	227 (17.6)	15 (14.6)	170 (17.5)	42 (19.5)
Never married but cohabiting	167 (12.9)	10 (9.7)	141 (14.5)	16 (7.4)
Married	564 (43.8)	47 (45.6)	405 (41.8)	112 (52.1)
Divorced or widowed	329 (25.6)	31 (30.1)	253 (26.1)	45 (21.0)
Highest education				
Elementary school or below	481 (37.4)	40(38.8)	369 (38.1)	72 (33.5)
Junior high school	582 (45.2)	45(43.7)	419 (43.2)	118 (54.9)
Senior High school or above	224 (17.4)	18(17.5)	181 (18.7)	25 (11.6)
Annual income (USD)				
< 2000	12 (0.9)	1 (1.0)	11 (1.14)	0 (0.0)
2001–5000	202 (15.7)	16 (15.5)	113 (11.7)	73 (34.0)
5001–9000	398 (30.9)	37 (35.9)	281 (29.0)	80 (37.2)
9001–$14 000	327 (25.4)	22 (21.4)	267 (27.6)	38 (17.7)
> 14 000	348 (27.0)	27 (26.2)	297 (30.7)	24 (11.1)
Employed status				
Self-employed	572 (44.4)	46 (44.7)	436 (45.0)	90 (41.9)
Employed by a boss	715 (55.6)	53 (55.3)	524 (55.0)	123 (58.1)
Length of time working in current location				
0–6 months	383 (29.8)	31 (30.1)	225 (23.2)	127 (59.1)
7–12 months	254 (19.7)	8 (7.8)	201 (20.7)	45 (20.9)
Over 1 year	650 (50.5)	64 (62.1)	543 (56.1)	43 (20.0)
Number of cities worked in for selling sex				
1	730 (56.7)	62 (60.2)	536 (55.3)	132 (61.4)
2	261 (20.3)	16 (15.5)	217 (22.4)	28 (13.0)
3	110 (8.6)	9 (8.7)	86 (8.9)	15 (7.0)
> 3	186 (14.5)	16 (15.5)	130 (13.4)	40 (18.6)
Residence				
Sampling province	662 (51.4)	60 (58.3)	480 (49.5)	122 (56.7)
Other province	625 (48.6)	43 (41.8)	489 (50.5)	93 (43.3)

*USD* United States dollar; *HIV*Human immunodeficiency virus

### Sexual behaviors

Of 1287 individuals, the median number of clients served in the past month was 22 (Interquartile range [IQR]: 12–56). The median amount of payment received for vaginal sex was USD 20 (IQR: 15–45). And 42.1% (542/1287) reported using condoms consistently when engaged in commercial vaginal sex in the past month. For oral sex, 58.3% (750/1287) reported providing oral sex in the past month, of whom only 16% (120/750) reported using condom consistently. For anal sex, 18.9% (243/1287) reported receiving anal sex in the past month and 41.6% (101/243) reported using condom consistently. The vast majority of women (78.4%, 1009/1287) reported having never used drugs before or during sex. Almost three-fifth of women (59.7%, 768/1287) reported bulk purchasing of condoms, and a small proportion of individuals (5.2%, 67/1287) have experience of bulk purchasing HIV self-testing kits (Table 2).

**Table 2:** Sexual behavior characteristics among female sex workers, a cross-sectional survey in China, 2019

Variables	Total	HIV testing, *n* (%)		
		Self-tester	Facility-tester	Non-tester
Total*	1287	103 (8.0)	969 (75.3)	215 (16.7)
Number of clients served in the past month, median (IQR)				
	22 (12–56)	20 (10–60)	25 (13–60)	20 (10–30)
Received money for vaginal sex (USD), median (IQR)				
	20 (15–45)	25 (15–45)	25 (15–45)	20 (15–30)
Consistently used condom in commercial vaginal sex in past month				
	542 (42.1)	39 (37.9)	418 (43.1)	85 (39.5)
Provided oral sex in the past month				
	750 (58.3)	68 (66.0)	588 (60.7)	94 (43.7)
Consistently used condom in commercial oral sex in past month				
	120 (16.0)	12 (17.7)	83 (14.1)	25 (26.6)
Provided anal sex in the past month				
	243 (18.9)	32 (31.1)	188 (19.4)	23 (10.7)
Consistently used condom in commercial anal sex in past month				
	101 (41.6)	13 (40.6)	77 (40.9)	11 (47.8)
Used drugs before or during sex				
	278 (21.6)	37 (35.9)	187 (19.3)	54 (25.1)
Injecting drugs in the past 6 months				
	56 (4.3)	8 (7.8)	43 (4.4)	5 (2.3)
Received any kind of HIV/STD-related services in the last year				
	1232 (95.7)	101 (98.1)	951 (98.1)	180 (83.7)
Bulk purchase of condoms				
	768 (59.7)	64 (62.1)	625 (64.5)	79 (36.7)
Bulk purchase of HIV self-test kits				
	67 (5.2)	22 (21.4)	36 (3.7)	9 (4.2)
Tested for other STIs in the past 6 months				
	646 (50.2)	77 (74.8)	522 (53.9)	47 (21.9)
Diagnosed with other STIs				
	394 (30.6)	38 (36.9)	320 (33.0)	36 (16.7)
Ever tested in the hospital				
	692 (53.8)	82 (79.6)	610 (62.9)	-
Ever tested in the community				
	794 (61.7)	65 (63.1)	729 (75.2)	-

*IQR* Interquartile range; *STI* Sexual transmitted infection

### HIV self-testing experience

Among the individuals who had self-tested for HIV, more than half reported that the self-test was their first ever HIV test (59.2%, 61/103). A minority of individuals reported ever giving a HIV self-test kit to a client (12.6%, 13/103) or selling a HIV self-test kit to a client (2.9%, 3/103). Approximately one-third of individuals reported an increase in HIV test frequency after their first use of HIV self-testing (30.1%, 31/103). The most common place to obtain a HIV self-test kit was from a community-based organization (75.7%, 78/103), followed by from an online vendor (22.3%, 23/103) (Table 3).

**Table 3:** Past HIV self-test experience, post-test health services utilization, and potential harms of HIV self-testing among Chinese female sex workers

Attributes	HIV self-tester (*n* = 103, %)
Characteristics of self-testing	103
Location where self-test kit was obtained	
Community-based organization	78 (75.7)
Online drug store	23 (22.3)
Hospital	21 (20.4)
Friend	13 (12.6)
Pharmacy	8 (7.8)
Self-testing results (last self-test)	
Reactive	8 (7.8)
Not sure	3 (2.9)
Negative	92 (89.3)
Post-test actions	11
Sought care following reactive/uncertain self-testing result	9 (81.8)
Time since reactive/uncertain self-testing result to seeking care	9
0–2 weeks	6 (66.7)
2–4 weeks	-
1–3 months	3 (33.3)
> 3 months	-
Location for seeking care	9
General hospital	2 (22.2)
Specialist STI service	3 (33.3)
Center for Disease Control and Prevention	2 (22.2)
Pharmacy/Online counseling/others	2 (22.2)
Benefits	103
Self-test as their first-time test	61 (59.2)
Gave a self-test kit to a client	13 (12.6)
Sold a self-test kit to a client	3 (2.9)
Increased testing uptake after first self-test	31 (30.1)
Adverse events	103
Self-testing influenced sex pricing negotiation	22 (21.4)
Police kept self-test kits as the evidence to accuse you of selling sex	3 (2.9)
Pressured self-testing	7 (6.8)
Types of pressure	103
Physical violence	1 (1.0)
Threats of violence	2 (1.9)
Verbal abuse	2 (1.9)
Psychological pressure	3 (2.9)
Excessive control of activities	2 (1.9)
Withholding of household resources	2 (1.9)
Threatening to end a relationship	3 (2.9)

*STI* Sexual transmitted infection; *HIV* Human immunodeficiency virus

A small proportion of individuals reported a reactive result in their last HIV self-test (7.8%, 8/103), or an indeterminate result (2.9%, 3/103). The majority sought care following reactive/indeterminate HIV self-testing result (81.8%, 9/11). Among those individuals who sought care after HIV self-testing, most women sought care within two weeks (66.7%, 6/9), and either at a specialty sexually transmitted infection (STI) clinic or in a health facility run by the Centers for Disease Control (Table 3).

Among individuals who had HIV self-tested, around one-fifth reported HIV self-testing results influenced the price of sex (21.4%, 22/103). A minority of individuals reported ever experiencing pressure to undertake HIV self-testing (6.8%, 7/103). The most common pressure was psychological pressure (2.9%, 3/103), followed by threatened to end a relationship (2.9%, 3/103) (Table 3).

### Difficulties and reasons for performing HIV self-testing

A majority of HIV self-testers reported some difficulties in performing HIV self-testing (71.84%, 74/103). Pricking fingers (59.5%, 44/74) or using collection tube to collect blood (50.0%, 37/74) were the most commonly reported difficulty. The most commonly reported reasons for using HIV self-testing were that they wanted to know their infection status (55.3%, 57/103), and they recently had high risk contact (54.4%, 56/103). The most common reason for not performing self-testing was that they had never heard of HIV self-testing before (42.6%, 504/1184) (Additional file 1: Table S1).

### Factors correlated with HIV self-testing

In the multivariable model adjusted for age, legal marital status, educational attainment and annual income, the following factors were positively correlated with HIV self-testing: receiving anal sex in the past month (adjusted odds ratio [a*OR*] = 2.2, 95% *CI* 1.4–3.5), using drug before or during sex (a*OR* = 2.8, 95% *CI* 1.8–4.5), injecting drugs in the past six months (a*OR* = 2.6, 95% *CI* 1.2–6.0), being diagnosed with other STIs (a*OR* = 1.6, 95% *CI* 1.0–2.5), tested for other STIs in the past 6 months (a*OR* = 3.4, 95% *CI* 2.1–5.5), ever tested in the hospital (a*OR* = 3.4, 95% *CI* 2.0–5.6), and ever tested in the community (a*OR* = 1.5, 95% *CI* 1.2–1.9) (Table 4).

**Table 4:** Factors correlated with HIV self-testing among Chinese female sex workers, 2019

Characteristics	HIV self-tester (*n* = 103)		
	*n* (%)	c*OR * (95% *CI*)	a*OR* (95% *CI*)^a^
Number of clients served in the past month, median (IQR)	20 (10–60)	1.000 (0.997–1.002)	0.999 (0.996–1.003)
Number of clients served in the past month			
≤ 30	65 (63.1)	*ref*	*ref*
31–60	15 (14.6)	0.8 (0.5–1.5)	0.7 (0.4–1.4)
61–90	6 (5.8)	0.7 (0.3–1.7)	0.6 (0.2–1.6)
> 90	17 (16.5)	1.3 (0.7–2.3)	1.3 (0.6–2.4)
Consistently used condom when engaged in commercial vaginal sex in past month			
Yes	39 (37.9)	0.8 (0.5–1.2)	0.9 (0.6–1.4)
No	64 (62.1)	*ref*	*ref*
Provided oral sex in the past month			
Yes	68 (66.0)	1.4 (0.9–2.2)	1.4 (0.9–2.4)
No	35 (34.0)	*ref*	*ref*
Consistently used condom when engaged in commercial oral sex in past month			
Yes	12 (17.6)	1.14 (0.6–2.2)	1.1 (0.5–2.2)
No	56 (82.4)	*ref*	*ref*
Provided anal sex in the past month			
Yes	32 (31.1)	2.1 (1.3–3.2)**	2.2 (1.4–3.5)*
No	71 (68.9)	*ref*	*ref*
Consistently used condom when engaged in commercial anal sex in past month			
Yes	13 (40.6)	0.9 (0.4–2.0)	0.9 (0.4–2.2)
No	19 (59.4)	*ref*	*ref*
Used drugs before or during sex			
Yes	37 (35.9)	2.2 (1.4–3.4)***	2.8 (1.8–4.5)***
No	66 (64.1)	*ref*	*ref*
Injected drugs in the past 6 months			
Yes	8 (7.8)	2.0 (0.9–4.3)	2.6 (1.2–6.0)*
No	95 (92.2)	*ref*	*ref*
Received any kind of HIV/STD-related services in the last year			
Yes	101 (98.1)	2.4 (0.6–9.8)	2.3 (0.6–9.8)
No	2 (1.9)	*ref*	*ref*
Bulk purchased condoms			
Yes	64 (62.1)	1.1 (0.7–1.7)	1.2 (0.7–1.8)
No	39 (37.9)	*ref*	*ref*
Tested for other STIs in the past 6 months			
Yes	77 (74.8)	3.2 (2.0–5.1)***	3.4 (2.1–5.5)***
No	26 (25.2)	*ref*	*ref*
Diagnosed with other STIs			
Yes	38 (36.9)	1.4 (0.9–2.1)	1.6 (1.0–2.5)*
No	65 (63.1)	*ref*	*ref*
Ever tested in the hospital			
Yes	82 (79.6)	2.7 (1.8–4.0)***	3.4 (2.0–5.6)***
No	21 (20.4)	*ref*	*ref*
Ever tested in the community			
Yes	65 (63.1)	1.5 (1.2–1.9)***	1.5 (1.2–1.9)**
No	38 (36.9)	*ref*	*ref*

*cOR* Crude odd ratio; *aOR* Adjusted odd ratio; *CI* Confidence interval; *IQR* Interquartile range

*P < 0.05; **P < 0.01; ***P < 0.001

^a^Multivariate logistic regression adjusted with age, legal marital status, educational attainment, monthly income

## Discussion

Female sex workers are at high risk of HIV acquisition and transmission [24]. Our study suggests that self-testing for HIV could expand overall testing uptake, increase testing frequency, and has limited potential harms among female sex workers. This study expands the literature by focusing on HIV self-testing among female sex workers, including women from multiple provinces, and exploring associated benefits and harms. Findings from this study can help inform HIV self-testing interventions among female sex workers.

Our study suggests that few female sex workers in China have performed HIV self-testing. This is consistent with rates of HIV self-testing reported in Malawi [14], Zimbabwe [15], Uganda [12], and Kenya [13]. Since the World Health Organization released guidelines recommending HIV self-testing among under-served and high-risk populations in 2016 [25], many studies in the sub-Saharan Africa have shown that HIV self-testing has a good acceptability and feasibility for female sex workers [12, 14, 15, 26]. Studies have suggested that adding HIV self-testing to existing community-based testing and counseling services among female sex workers is acceptable, cost-effective and efficient to improve linkage to care [15, 26, 27]. China should take more effort to explore strategies integrating HIV self-testing to existing facility-based testing services. Additionally, in the context of wide online availability of HIV self-testing kits in China [11], our data could help to inform interventions.

Our study found low frequency of physical violence or other types of violence related to HIV self-testing among female sex workers. The frequency of physical violence associated with HIV self-testing is comparable to that reported in Zambia [15] and Kenya [13]. Intimate partner violence is common and could be exacerbated by self-testing services that inadvertently allow a sex worker manager or partner to influence testing behaviors [13, 28]. Our results suggest that self-testing is not associated with physical violence, although further research and attention are needed.

We found that self-testing for HIV can effectively reach high-risk female sex workers, and facilitate higher frequency testing. In our study, HIV self-testing was correlated with receiving commercial anal sex in the past month and drug use before or during sex. This finding is consistent with studies conducted among men who have sex with men in China [21] and France [29]. Additionally, our study found that a large proportion of HIV self-testers reported having never been tested for HIV before the self-testing, and approximately one-third of HIV self-testers reported increasing testing uptake after initial use of HIV self-testing. This suggests HIV self-testing has the potential to increase the frequency of HIV testing among female sex workers, specifically among individuals not reached by provider-based strategies [11, 30].

Our study has several limitations. First, the survey captured a convenience sample of female sex workers population in China, likely resulting in selection bias. Although the convenience sampling method can generally be implemented more easily, faster and with fewer resources [31], the study sample might not be representative of the population as a whole, which limits the statistical inference and generalizations [32]. Second, this study was conducted among female sex workers in cities with relatively high involvement in HIV prevention programs. The results of this study may not be generalizable to female sex workers in cities that have fewer HIV prevention programs. Third, all the data were collected through self-report, which may be prone to information bias.

Although self-testing for HIV may be effective in expanding female sex workers testing uptake and frequency, several challenges remain. Individuals with a reactive result require a clinic visit for confirmation of infection, which can be inconvenient, and create a barrier for female sex workers to linkage to care [33]. Female sex workers tend to be high degree of mobility and vulnerable to criminality [34], highlighting the need of social support for training, counseling, and ancillary self-testing services [15]. Adequate linkages to counseling, treatment, and care for HIV self-testers who test positive, and test quality assurance remain essential [35].

## Conclusions

Innovative approaches are needed to improve HIV testing uptake among female sex workers. Our findings suggest that HIV self-testing has the potential to expand overall testing uptake, enable more frequent testing, reach sub-groups of high-risk female sex workers, and has limited potential harms among female sex workers. Self-testing for HIV should be incorporated among Chinese female sex workers as a complement to facility-based testing services. Future studies to explore effective mode of promoting HIV self-testing among female sex workers are warranted.

## Supplementary information

**Additional file 1: Table S1**. Difficulties and reasons for performing HIV self-testing among Chinese female sex workers.

## Data Availability

The datasets used and/or analyzed during the current study are available from the corresponding author on reasonable request.

## Abbreviations

HIV: Human immunodeficiency virus
STI: Sexually transmitted infections
LMIC: Low- and middle-income countries
*CI*: Confidence interval
c*OR*: Crude odd ratio
a*OR*: Adjusted odd ratio
IQR: Interquartile range
