# Supplementary material for: Correlates of HIV self-testing among female sex workers in China: implications for expanding HIV screening
Source: Infect Dis Poverty. 2020 Oct 22;9:147. doi: 10.1186/s40249-020-00765-5 (PMC7583185; doi:10.1186/s40249-020-00765-5)
Supplement: Supplementary file 1 — Additional file 1: Table S1. Difficulties and reasons for performing HIV self-testing among Chinese female sex workers. [file 40249_2020_765_MOESM1_ESM.docx]

| **Table S1 Difficulties and reasons for performing HIV self-testing among Chinese female sex workers** | |
| --- | --- |
| **Items** | **HIV self-testing**  **(n, %)** |
| **Difficulties in performing self-testing** |  |
| Pricking finger | 44(59.5) |
| Squeezing the finger firmly to extract blood | 18(24.3) |
| Using collection tube to collect blood | 37(50.0) |
| Understanding the instructions for performing the test | 23(31.1) |
| Timing the test | 13(17.6) |
| Removing lancet cap | 13(17.6) |
| Interpreting the results | 12(16.2) |
| **Total** | 74 |
| **Reasons for performing self-testing?** |  |
| I wanted to know my infection status | 57(55.3) |
| I want to be the first person to read my test result. | 35(34.0) |
| I had symptoms and I was worried of infection | 39(37.9) |
| I recently had high risk contact | 56(54.4) |
| I was recommended to test by healthcare staff | 27(26.2) |
| My regular partner asked me to test | 10(9.7) |
| A casual partner asked me to test | 11(10.7) |
| **Total** | 103 |
| **Reasons for not using self-testing?** |  |
| Don’t know where to obtain self-test kit | 458(38.7) |
| Haven’t heard of self-testing | 504(42.6) |
| Don’t familiar with instruction guideline | 296(25.0) |
| Have already tested at a clinic | 350(29.6) |
| Worried of giveaway personal information | 265(22.4) |
| Don’t believe the result | 347(29.3) |
| Worried about blood collection | 268(22.6) |
| Worried about not able to interpret the results | 306(25.8) |
| Worried about the cost | 99(8.4) |
| **Total** | 1184 |
